# Can Physical Exercise Improve the Residents' Health?

**DOI:** 10.3389/fpubh.2021.707292

**Published:** 2021-06-29

**Authors:** Cong Li, Jin-Hong Chen, Xi-Hua Liu, Shu-Qi Ren

**Affiliations:** ^1^School of Economics, Qingdao University, Qingdao, China; ^2^School of Economics, Central University of Finance and Economics, Beijing, China

**Keywords:** BMI, heterogeneity test, physical exercise, residential health level, CFPS

## Abstract

With the rapid development of China's economy and society, the quality of life of residents has continued to improve, and the concept of health has gradually taken root in the hearts of the people. This paper uses the 2018 data of the China Family Panel Studies (CFPS) to analyze the internal mechanism and influencing factors of participating in physical exercise on the health level of residents. The results show that participating in physical exercise can significantly improve the subjective and objective health level of residents, and the result passed two important robustness tests. Heterogeneous test shows that the degree of influence of participation in physical exercise on the health level of residents varies according to the different regional and individual characteristics; the effect of urban residents' physical exercise to improve their health level is better than that of rural residents; and the effect of physical exercise on the improvement of health level is more obvious in the eastern and western areas, those who have not completed compulsory education, and those with high incomes. This study will have strong guiding significance for the improvement of the overall level of national health in China and will provide a theoretical basis and guiding opinions for the construction of healthy China.

## Introduction

Health is a prerequisite for human survival and development, and it is the most fundamental basis for human productive life. With the continuous improvement of people's living standards, health has been paid more and more attention, and the state every year to improve the national health level of a large amount of investment, in the field of health planning, is also improved year by year. The Fourth Plenary Session of the 19th Central Committee of the Communist Party of China proposed that we should strengthen the protection of the health system, pay attention to the whole process of life cycle and health, and ensure that the masses enjoy continuous and fair health services.

The Fifth Plenary Session of the 19th Central Committee adopted “the Outline of the 14th 5-Year Plan for National Economic and Social Development and the 2035 Vision Goals” (hereinafter referred to as the “Outline”), “Outline” states that party should continue to deepen the construction of a healthy China, improve health promotion policies, accelerate the development of healthy industries, and promote the quality of the people and the level of social civilization to a new height. To this end, the National Health and Wellness Commission has made every effort to promote the construction of health in China and promulgated and implemented a series of policies to promote a disease-centered to health-centered transformation, in which “the basic health care and health promotion act” provides for the integration of health education into the national education system in order to safeguard citizens' right to health. It can be seen that the Party and the government have always paid close attention to the health problems of residents, so it is particularly important to study in depth the factors affecting the health level of residents.

The World Health Organization defines health as “a state of physical, mental and social well-being,” which means not only the absence of a disease but also the healthy development of the body and mind, and the harmony of family and social life. Physical education is one of the effective ways to improve physical fitness and mental health in terms of health. Research shows that physical activity can control and reduce health hazards, establish and maintain a beneficial health environment, and play a “first-level prevention” effect (1). Physical activity enhances physical fitness by improving physiological function, affecting the body's morphological structure and athletic ability (2), impacting mental health by promoting intelligent development, improving emotional control, and enhancing social resilience (3).

It is well-known that medical treatment is a passive measure to obtain health after suffering from a disease; in contrast, physical education is a proactive approach to health intervention, which emphasizes active health, “cure the disease before illness” (4).

From the national point of view, in order to enhance people's physical fitness and improve health level, the state actively carried out the “National Fitness Program,” and during the “12th 5-Year Plan” period, it has initially formed a relatively sound public service system for national fitness. During the 13th 5-Year Plan period, the national fitness service system was more perfect, and the awareness of mass fitness was widely enhanced. The Outline calls for the continuation of in-depth national fitness campaign, the construction of sports facilities according to local conditions, the expansion of sports consumption, and at the same time the development of sports industry, so as to achieve the goal of sports power. The Outline of “Healthy China 2030” also points out that the construction of sports facilities should be strengthened and that the scientific and rational development of sports activities by key groups should be promoted. It can be seen that sports has risen to the height of national strategy, and its role in the health of residents cannot be ignored.

The rest of the paper is structured as follows: the second part is literature review and research hypotheses; the third part is data selection, variable description, and model setting; the fourth part is empirical analysis, including benchmark regression, robustness test, and heterogeneity test; and the fifth part is research conclusions and enlightenment.

## Literature Review and Research Hypothesis

For the study of the health status of residents, the literature has been carried out from two perspectives: the first category is mostly concerned about the individual situation of residents, including residents' income, education level, socioeconomic status, and other factors. The second category is mostly concerned with external factors, including government healthcare, environment, urbanization, and other factors.

For the residents' income factor, the scholars hold different opinions on this issue. Qi (5) pointed out that income affects health by improving living standards and medical conditions and that there are urban–rural and occupational differences. Ren et al. (6) and Wang and Xu. (7) held different views; they think that at the low income level, increasing income has a positive effect on residents' health self-assessment, and at the high income level: the former thought that income increase has a negative impact on health, while the latter thought that the impact is not obvious. Su et al. (8) further suggested that the health impact of income should also take into account regional and social differences (8).

For the education level factor, most scholars use micro-databases for factor analysis and put forward more consistent conclusions. Cheng et al. (9) used China's elderly health factors tracking survey data, through channel analysis, to find out that education has a role in promoting health. Hu (10) and Li et al. (11) showed that there are urban and rural and gender differences in the impact of education on health, while Zhao and Hu (12) used data from the China Family Panel Studies (CFPS) 2010 to analyze that education does not necessarily promote health, primary education promotes health by raising income, and higher education has a negative impact on health by affecting residents' sleep time and frequency of phone calls. Huang et al. (13), Shen and Zhu (14), and Yuan et al. (15), using China Health and Nutrition Survey (CHNS) and CFPS data research, all thought that the promotion of socioeconomic status is conducive to promoting health level; Huang et al. (16) believed that higher socioeconomic status by improving the frequency of residents' sports and leisure, insurance participation rate to improve the health level, and the promotion of socioeconomic status will also improve risk asset participation and reduce neighborhood trust, thereby reducing the level of health. Overall, the positive effect is greater than the negative effect (16).

For the external factors, studies have shown that it will also have varying degrees of impact on the health of residents. Pan et al. (17) used the data of the State Council's basic medical insurance pilot assessment of urban residents to study the relationship between medical insurance and health, and the results showed that medical insurance is beneficial to the health of the insured residents and has a greater impact on people with poor socioeconomic status. Using macro data research (17), Li and Yu (18) confirmed that government health spending has significantly improved the health of residents. Further, Li et al. (19) measured the scale of health input from two angles on the basis of micro data, and they found that the effect of personal health expenditure is better than that of government and social health expenditure, both from the perspective of per capita health expenditure and the total amount of health expenditure. Jia et al. (20) found that the higher the forest cover, the lower the mortality rate of cancer, stomach cancer, and other diseases. Wang et al. (21) and Chen (22) found that the expansion of urban green space area affected the overall health of residents, physiological health, and mental health in three levels of health promotion. Zhao et al. (23), Chang and Zhong (24), and Chen (25) used a combination of empirical models and theoretical analysis to study the positive impact of urbanization on health, i.e., the improvement of urbanization level to promote the health level of residents, and found that the impact of regional differences and the effect on the health level of the eastern area are greater than those of the central and western areas.

For the study of the relationship between physical education and residents' health level, the existing literature mainly starts from the perspective of objective health and subjective health and holds that participation in physical exercise has a positive effect on the health level of residents.

From an objective health perspective, Zhang (26) believed that sports is an important way for national fitness, enhancing physical fitness, but also promoted sports to become an important “lifestyle” content; and Wang and Xu (7) also confirmed that with healthy lifestyle, the health rate of the residents who exercise is higher, and the unhealthy rate is lower. Wang and He (27) and Ding et al. (28) pointed out that the occurrence of many chronic diseases and lack of physical activity are closely related and that moderate physical exercise for the prevention and intervention of chronic diseases has a positive role. Liu and Yu (2) and Xia et al. (29) believed that the current static lifestyle has a negative impact on health and that physical activity is insufficient to lead to an increase in the incidence of many lifestyle diseases. Sports improve health by effectively enhancing physical function and regulating mood. Therefore, we should advocate moderate sports and take the method of exercise intervention to guide the national fitness, in order to create a healthy life. Lu (4) analyzed that sports through active ways to improve the health of individuals should strengthen the integration of sports and other fields. Scholars such as An and Wang (30), Li et al. (31), and Miu and Bian (32) used fixed-effects models to confirm that physical exercise has a significant positive impact on health levels and that residents with high participation in physical exercise have higher health self-assessment than those with low participation.

From the perspective of subjective health, Bai et al. (3) and Sun et al. (33) pointed out that sports improve mental health by improving cognitive function, improving self-perception, and eliminating fatigue and depression. Wang et al. (34) and Dai (35) used questionnaires to draw conclusions that physical exercise is beneficial to improving the mental health status of college students. In view of the research on the mental health status of primary and secondary school and the elderly population, Lan and Luo (36) used the investigation and analysis of the mental health status of primary and secondary school students in some areas to find out that sports can improve students' psychological quality level by adjusting students' emotions to relieve physical and mental contradictions and release learning pressure, so as to promote mental health; Yin et al. (37) further put forward that the motivation to participate in sports activities, the degree of fatigue after activities, and the external motivation to participate in sports activities directly affect the mental health of primary and secondary school students.

In summary, the existing literature points out the possible relationship between physical activity and residents' health level and expounds that physical exercise improves health level from both objective health and subjective health. In the objective health perspective, the lack of physical activity leads to an increase in the incidence of chronic diseases, static lifestyle is not conducive to health, and people can enhance physical fitness through active exercise, so as to obtain health. From a subjective health perspective, physical exercise improves mental health by improving cognition and venting emotions and promotes the health of people of different ages. The existing literatures mainly focus on qualitative analysis instead of quantitative analysis, and the data sources mostly use regional population questionnaires, lacking effective support from macroscopic national data. In addition, most literature analyses are elaborated from the subjective and objective aspects, and there is a lack of studies integrating the two dimensions.

For this, based on the CFPS data in 2018, taking part in physical exercise with the ordinary least squares (OLS) and tobit model influence on residents' health level, the study found that participation in physical exercise has significant positive influence on residents' health level and affects the physical and mental health in the process of the individual character differences and regional differences. This is of theoretical reference significance to comprehensively improve the national health level of China.

Based on the above discussion, this paper proposes the following research hypothesis:

**Hypothesis:** Participation in physical exercise has a positive impact on the health level of residents. The higher the frequency of participation in physical exercise, the better the health status.

The contribution margin of this paper mainly includes the following two aspects: first, there is an innovation in data selection in this paper, and it uses the data of the CFPS to carry out a quantitative analysis on the impact of physical exercise on the health level of residents, which covers a wide range of data and ensures the reliability of the results. Second, there is an innovation in the selection of variable dimensions, and the objective and subjective health of residents are taken into account to measure the health level, and the health status of residents is evaluated more comprehensively.

## Data Sources and Specification of Variables

### Data Sources

The data used in this paper are from the 2018 CFPS. The CFPS was carried out by China Social Science Survey Center of Peking University. The survey sample covers 25 provinces/municipalities/autonomous regions, including individual, household, and community data. The questionnaire covers various indicators reflecting residents' family relationship, living environment and economic situation, residents' personal education status, occupation status, lifestyle, pension, and medical care, so as to measure residents' health status comprehensively. In order to ensure the validity of the results, invalid data such as “don't know,” “refuse to answer,” inapplicable, and blank were deleted, and 10,543 valid sample data were finally obtained.

### Specification of Variables

#### Explained Variables

The health level of residents reflects their physical, mental, social, and economic status and security services. International practice generally uses average life expectancy, infant mortality rate, and maternal mortality rate to measure the health level of residents. In the medical field, various disease indicators are generally used to measure the health level of residents. Considering the applicability and availability of data, this paper selects residents' own health evaluation as a proxy index to measure residents' health level. The details are shown in Table 1.

**Table 1:** Variable definition and assignment.

	**Variable**	**Variable assignment**
Explained variables	Physical condition	Very healthy = 1; health = 2; relatively healthy = 3; general = 4; unhealthy = 5
Explanatory variables	Exercise frequency	Frequency (times)
Control variables	Rural types	Town = 1, country = 0
	Gender	Male = 1, female = 0
	Age	Age (years)
	Marriage status	Married = 1, unmarried = 0
	Party membership	Yes = 1, no = 0
	Education level	Illiterate or semi-literate = 1; primary school = 2; junior high school = 3; high school/technical secondary school/technical school/vocational high school = 4; college degree = 5; bachelor's degree = 6; master = 7; doctorate = 8
	Happiness level	From 0 to 10, happiness goes up
	Hospitalization experience	Yes = 1, no = 0
	Chronic disease	Yes = 1, no = 0
	Medical insurance	Yes = 1, no = 0
	Household income	Take the logarithm of annual household income (yuan)
	Household expenditure	Take the logarithm of annual household expenditure (yuan)
	Family size	Number of family size (persons)
	Healthcare expenditure	Take the logarithm of healthcare spending
	Neighbor relations	From 0 to 10, trust goes up
	Gift expenditure	Yes = 1, no = 0

#### Core Explanatory Variables

In this paper, the frequency of physical exercise participation is used to measure the degree of physical exercise participation. The ordered discrete variable is assigned 0–10 to represent the number of times of physical exercise participation. Due to the wide range of original data values, in order to ensure the robustness of regression results, the “exercise frequency” was reduced by 1%.

#### Control Variables

In terms of personal characteristics, in addition to the core explanatory variables, the existing literature was referred to (7, 12, 19), selecting respondents' rural types, gender, age, marriage status, party membership, education level, and happiness level as the control variables at the individual level.

At the level of family characteristics, the paper selects the household income, household expenditure, family size, healthcare expenditure, and medical insurance as the control variables. Among them, due to the large numerical span of household income, household expenditure, and medical care expenditure and in order to avoid biasing the regression results, and the logarithm of their data was taken as the final variable. In terms of community characteristics, neighbor relations and gift expenditure were selected as the control variables. Descriptive statistics of variables are shown in Table 2.

**Table 2:** Descriptive statistics of variables.

**Variable**	**Observation**	**Mean**	**Standard deviation**	**Minimum**	**Maximum**
Physical condition	10,543	3.143	1.215	1	5
Exercise frequency	10,543	2.603	3.113	0	10
Rural types	10,543	0.503	0.500	0	1
Gender	10,543	0.500	0.500	0	1
Age	10,543	49.99	14.39	22	81
Marriage status	10,543	0.831	0.375	0	1
Party membership	10,543	0.00645	0.0801	0	1
Education level	10,543	2.714	1.329	1	8
Happiness level	10,543	7.303	2.235	0	10
Hospitalization experience	10,543	0.140	0.347	0	1
Chronic disease	10,543	0.193	0.394	0	1
Medical insurance	10,543	0.918	0.274	0	1
Household income	10,543	10.47	1.408	0	14.51
Household expenditure	10,543	10.11	1.174	0	14.43
Family size	10,543	3.585	1.869	1	17
Healthcare expenditure	10,543	6.927	2.785	0	12.87
Neighbor relations	10,543	6.733	2.120	0	10
Gift expenditure	10,543	0.895	0.307	0	1

The value of health status from 1 to 5 represents very healthy to unhealthy, with an average value of 3.143, indicating that most of the residents in the sample are relatively healthy. The value range of exercise frequency is 0–10, and the mean value is 2.603, indicating that the overall exercise frequency of residents within the sample range is not high. The mean value of urban and rural types is 0.503, indicating that the distribution of urban and rural areas in the sample is relatively uniform. The mean of gender is 0.500, indicating the same number of men and women in the sample. The age range is 22–81, and the mean value is 49.99, indicating that most residents in the sample range are in the middle age stage.

### Model Specification

The least square method is the most commonly used method to solve curve fitting problems. Its basic assumptions are that the mean value of residuals is 0, the variance is constant, the residuals are statistically independent of each other, and the residuals are normally distributed and independent of variables. It minimizes the sum of squares of the difference between the estimated value of the explained variable and the actual observed value in order to fit the sample observations as much as possible. The model is set as follows:

(1) $$\begin{array}{r} hea_{i}=\alpha_{0}+\alpha_{1}exe_{i}+\gamma X_{i}+\delta_{i} \end{array}$$

*hea*_*i*_ is the explanatory variable, representing the health status of individual *i*; *exe*_*i*_ is the explanatory variable, representing the frequency of individual *i* participating in physical exercise; *X*_*i*_ represents a series of control variables mentioned above on individual, family, and social levels; α_0_ is a constant term; and α_1_ and γ are coefficient vectors. Among them, α_1_ is the coefficient that this paper focuses on. δ_*i*_ is a random disturbance term.

A tobit model is also called truncated regression model and censored regression model, and its application scenario is that the explained variables are roughly continuously distributed on positive values, but it includes some observed values with positive probability of 0. For example, when the dependent variable is >0, it is observed, and when it is ≤0, it is truncated at 0. In the tobit model, the meaning of the regression coefficient depends on the practical application, and the likelihood ratio test is used to test the regression coefficient. The explained variable in this paper is the truncated variable, so it is applicable to the model. The model is set as:

(2) $$\begin{array}{r} hea_{i}=\max\left(0,\;\beta exe_{i}+\;\varepsilon_{i}\right) \end{array}$$

*hea*_*i*_ is the health status of individual *i*, *exe*_*i*_ is the frequency of individual *i* participating in physical exercise, β is the coefficient vector, and ε_*i*_ is the error term.

## Empirical Analysis

### Benchmark Regression

Result (1) in Table 3 is the regression result of the OLS model with only core explanatory variables and regional fixed effects added. The results show that exercise frequency is negatively correlated with health status, and the increase in exercise frequency reduces the fitness level. Result (2) is the regression result of adding all control variables and excluding regional fixed effects. It can be seen that after adding all control variables, exercise frequency has a significant positive effect on health status. The higher the frequency of exercise, the stronger the physical fitness and the better the health. This provides empirical support for the research hypothesis of this paper.

**Table 3:** Benchmark regression.

**Variable**	**OLS**			**Tobit**		
	**(1)**	**(2)**	**(3)**	**(4)**	**(5)**	**(6)**
Exercise frequency	0.002	−0.019^***^	−0.019^***^	0.003	−0.028^***^	−0.027^***^
	(0.59)	(−5.52)	(−5.30)	(0.48)	(−5.66)	(−5.46)
Rural types		0.040^*^	0.026		0.044	0.029
		(1.72)	(1.11)		(1.35)	(0.86)
Gender		−0.203^***^	−0.207^***^		−0.290^***^	−0.294^***^
		(−9.52)	(−9.58)		(−9.55)	(−9.57)
Age		0.015^***^	0.015^***^		0.021^***^	0.021^***^
		(17.06)	(16.57)		(15.96)	(15.58)
Marriage status		0.015	0.020		−0.005	0.001
		(0.48)	(0.65)		(−0.12)	(0.01)
Party membership		−0.100	−0.121		−0.099	−0.128
		(−0.76)	(−0.92)		(−0.53)	(−0.69)
Education level		−0.001	0.004		−0.001	0.005
		(−0.12)	(0.35)		(−0.09)	(0.34)
Happiness level		−0.084^***^	−0.084^***^		−0.119^***^	−0.119^***^
		(−16.94)	(−16.79)		(−16.58)	(−16.51)
Hospitalization experience		0.484^***^	0.481^***^		0.761^***^	0.758^***^
		(15.10)	(14.98)		(16.14)	(16.06)
Chronic disease		0.671^***^	0.676^***^		1.003^***^	1.007^***^
		(23.65)	(23.70)		(24.15)	(24.17)
Medical insurance		0.021	0.032		0.035	0.048
		(0.55)	(0.81)		(0.64)	(0.86)
Household income		−0.035^***^	−0.037^***^		−0.051^***^	−0.053^***^
		(−3.80)	(−3.94)		(−3.82)	(−3.89)
Household expenditure		0.007	0.000		0.003	−0.004
		(0.60)	(0.03)		(0.20)	(−0.26)
Family size		−0.015^**^	−0.015^**^		−0.023^***^	−0.023^**^
		(−2.40)	(−2.27)		(−2.62)	(−2.44)
Healthcare expenditure		0.048^***^	0.049^***^		0.066^***^	0.068^***^
		(11.78)	(12.11)		(11.52)	(11.83)
Neighbor relations		−0.022^***^	−0.020^***^		−0.030^***^	−0.027^***^
		(−4.34)	(−3.93)		(−3.98)	(−3.64)
Gift expenditure		0.028	0.038		0.027	0.036
		(0.77)	(1.05)		(0.53)	(0.69)
_cons	2.810^***^	3.045^***^	3.067^***^	2.778^***^	3.241^***^	3.281^***^
	(22.89)	(23.95)	(17.90)	(16.06)	(17.82)	(13.53)
Regional fixed effects	Yes	No	Yes	Yes	No	Yes
N	10,543	10,543	10,543	10,543	10,543	10,543
R-squared	0.009	0.218	0.224			

^***^ and ^**^ respectively indicate significance at the 1and 5 levels.

Among the control variables, gender, age, hospitalization experience, chronic disease, household income, healthcare expenditure, neighbor relations, and happiness levels all have significant effects on health. Compared with women, men are in better health. It may be because men love sports more than women and therefore have better physical fitness. The older you are, the lower your physical fitness and the greater the possibility of suffering from various diseases, so your health becomes worse. Hospitalization experience is significantly negatively correlated with health status. This control variable indicates that the body has had a problem recently, and the possibility of not being cured or getting sick again is more likely, and the health status is worse. Chronic diseases are negatively correlated with health status. The fewer the chronic diseases, the healthier the body. A higher total family income means that the family's economic status is better, so its medical and healthcare capabilities are stronger, and therefore, its health status is better. Medical care expenditures are negatively correlated with health status. It may be that the more medical care expenditures, the poorer the health status itself, and the expenditures on healthcare have not played an effective role. The higher the trust in neighbors, the more harmoniously the neighbors get along, the more harmonious the relationship, the less pressure brought by the interpersonal relationship, and the better the health. The degree of happiness is based on the evaluation of residents' self-perception, which has a significant positive effect on health. The happier the residents, the more beneficial to physical and mental health and the better their health.

As far as the remaining control variables are concerned, the health status of urban residents is worse than that of rural residents. It may be that the development of urban modernization has led to a fast pace of life and high pressure to go to work, so the health level has decreased. In terms of marriage, the health status of married people is worse than that of unmarried people. It may be that after marriage, in addition to going to work, they also need to maintain family relationships and raise children. Compared with unmarried people, they have to bear more responsibilities and pressures, so their health status is relatively poor. Party membership is positively correlated with health level. Education level positively affects health status, indicating that the more you receive higher education, the more you will pay attention to your own health status, and the better your health status. The total annual family expenditure is negatively correlated with health. The more daily expenses, the less expenditure that can be spent on healthcare and health, so the health status is worse. The larger the family size, the larger the family population, the greater the economic burden, the smaller the expenditure on health, and the worse the health status.

Result (3) is the regression result after adding the regional fixed effects on the basis of result (2), which is basically consistent with result (2). Results (4)–(6) are the results of regression using the tobit model, which are basically the same as the results of the OLS model regression, except that the magnitude of the impact of the two is slightly different. This shows that the model and the benchmark regression results have a certain degree of robustness.

### Robustness Test

#### Replace Explanatory Variables

In order to test the robustness of the regression results, the 0–1 dummy variable is used to measure whether there is physical exercise instead of exercise frequency to measure physical participation. The value of physical exercise is 1, and the value of no physical exercise is 0. Result (1) is the OLS regression result with only the core explanatory variables, and the regional fixed effects are added. Result (2) is the regression result with all the control variables, and the regional fixed effects are added; and result (3) is the regression result of adding all control variables and including regional fixed effects. Results (1)–(3) show that participation in physical exercise is positively correlated with residents' health status, which shows that physical exercise promotes the improvement of people's health. Results (4)–(6) are the results of regression using the tobit model, which are basically consistent with the results of the OLS model regression, indicating that participation in physical exercise has a significant role in promoting the health of residents. The higher the frequency of participation in exercise, the better the health status. The results strongly verify the robustness and reliability of the regression results. The results are shown in Table 4.

**Table 4:** Robustness test (replace explanatory variables).

**Variable**	**OLS**			**Tobit**		
	**(1)**	**(2)**	**(3)**	**(4)**	**(5)**	**(6)**
Exercise or not	−0.060^**^	−0.129^***^	−0.126^***^	−0.085^**^	−0.185^***^	−0.180^***^
	(−2.52)	(−5.95)	(−5.79)	(−2.48)	(−5.96)	(−5.79)
Rural types		0.042^*^	0.028		0.047	0.031
		(1.82)	(1.19)		(1.44)	(0.93)
Gender		−0.203^***^	−0.207^***^		−0.290^***^	−0.294^***^
		(−9.53)	(−9.58)		(−9.56)	(−9.57)
Age		0.015^***^	0.015^***^		0.020^***^	0.020^***^
		(16.97)	(16.49)		(15.83)	(15.46)
Marriage status		0.012	0.018		−0.009	−0.003
		(0.39)	(0.57)		(−0.21)	(−0.07)
Party membership		−0.091	−0.113		−0.086	−0.116
		(−0.69)	(−0.86)		(−0.46)	(−0.63)
Education level		0.002	0.007		0.004	0.009
		(0.23)	(0.67)		(0.25)	(0.65)
Happiness level		−0.084^***^	−0.084^***^		−0.120^***^	−0.120^***^
		(−16.97)	(−16.82)		(−16.62)	(−16.55)
Hospitalization experience		0.483^***^	0.481^***^		0.759^***^	0.757^***^
		(15.09)	(14.98)		(16.12)	(16.05)
Chronic disease		0.673^***^	0.677^***^		1.005^***^	1.009^***^
		(23.70)	(23.75)		(24.20)	(24.22)
Medical insurance		0.020	0.031		0.034	0.047
		(0.52)	(0.80)		(0.61)	(0.85)
Household income		−0.035^***^	−0.037^***^		−0.051^***^	−0.052^***^
		(−3.75)	(−3.91)		(−3.78)	(−3.87)
Household expenditure		0.007	0.000		0.003	−0.005
		(0.59)	(0.01)		(0.19)	(−0.28)
Family size		−0.015^**^	−0.015^**^		−0.024^***^	−0.022^**^
		(−2.42)	(−2.27)		(−2.63)	(−2.44)
Healthcare expenditure		0.048^***^	0.049^***^		0.066^***^	0.068^***^
		(11.81)	(12.14)		(11.56)	(11.86)
Neighbor relations		−0.023^***^	−0.020^***^		−0.030^***^	−0.027^***^
		(−4.35)	(−3.94)		(−3.99)	(−3.65)
Gift expenditure		0.028	0.039		0.028	0.037
		(0.79)	(1.09)		(0.54)	(0.72)
_cons	2.854^***^	3.059^***^	3.092^***^	2.838^***^	3.263^***^	3.318^***^
	(23.15)	(24.10)	(18.08)	(16.34)	(17.97)	(13.70)
Regional fixed effects	Yes	No	Yes	Yes	No	Yes
N	10,543	10,543	10,543	10,543	10,543	10,543
R-squared	0.010	0.218	0.224			

^***^, ^**^, and ^*^ respectively indicate significance at the 1, 5, and 10% levels.

#### Replace the Explained Variable

According to international practice, the body mass index (BMI) is commonly used to assess the nutritional status of the human body and to measure the degree of body weight and health. Therefore, this paper uses the BMI (weight divided by the height squared) to replace the health status in measuring the health level of residents so as to verify the robustness of the regression results. The BMI is a continuous variable and is divided according to the World Health Organization international standards. Assign the value 18.5 < BMI < 30 = 1, BMI < 18.5, or BMI > 30 = 0. At this time, the regression result is still significant at the level of 1%, which indicates that the higher the frequency of participating in physical exercise, the higher the BMI and the higher the health level of residents. The research hypothesis of this paper has been verified once again. The results are shown in Table 5.

**Table 5:** Robustness test (replace the explained variable).

**Variable**	**OLS**			**Tobit**		
	**(1)**	**(2)**	**(3)**	**(4)**	**(5)**	**(6)**
Exercise frequency	0.003^***^	0.003^***^	0.003^***^	0.003^***^	0.003^***^	0.003^***^
	(3.22)	(3.08)	(3.10)	(3.22)	(3.08)	(3.10)
Rural types		0.007	0.008		0.007	0.008
		(1.16)	(1.23)		(1.16)	(1.24)
Gender		0.016^***^	0.018^***^		0.016^***^	0.018^***^
		(2.78)	(2.94)		(2.78)	(2.94)
Age		0.000	0.000		0.000	0.000
		(1.26)	(1.23)		(1.26)	(1.23)
Marriage status		0.036^***^	0.034^***^		0.036^***^	0.034^***^
		(4.21)	(3.93)		(4.21)	(3.94)
Party membership		−0.013	−0.013		−0.013	−0.013
		(−0.35)	(−0.36)		(−0.35)	(−0.36)
Education level		0.005^*^	0.005^*^		0.005^*^	0.005^*^
		(1.67)	(1.65)		(1.67)	(1.65)
Happiness level		−0.003^**^	−0.003^**^		−0.003^**^	−0.003^**^
		(−2.06)	(−2.43)		(−2.06)	(−2.43)
Hospitalization experience		−0.011	−0.012		−0.011	−0.012
		(−1.23)	(−1.29)		(−1.23)	(−1.30)
Chronic disease		−0.020^***^	−0.021^***^		−0.020^***^	−0.021^***^
		(−2.59)	(−2.71)		(−2.59)	(−2.71)
Medical insurance		0.002	0.001		0.002	0.001
		(0.17)	(0.06)		(0.17)	(0.06)
Household income		0.010^***^	0.010^***^		0.010^***^	0.010^***^
		(3.80)	(3.86)		(3.81)	(3.87)
Household expenditure		0.005	0.006^*^		0.005	0.006^*^
		(1.49)	(1.76)		(1.49)	(1.77)
Family size		−0.001	−0.001		−0.001	−0.001
		(−0.76)	(−0.33)		(−0.76)	(−0.33)
Healthcare expenditure		0.001	0.001		0.001	0.001
		(0.91)	(0.82)		(0.91)	(0.82)
Neighbor relations		0.001	0.001		0.001	0.001
		(0.74)	(0.50)		(0.74)	(0.50)
Gift expenditure		0.021^**^	0.018^*^		0.021^**^	0.018^*^
		(2.09)	(1.77)		(2.09)	(1.78)
_cons	0.921^***^	0.668^***^	0.686^***^	0.921^***^	0.668^***^	0.686^***^
	(30.24)	(18.88)	(14.37)	(30.29)	(18.90)	(14.40)
Regional fixed effects	Yes	No	Yes	Yes	No	Yes
N	10,543	10,543	10,543	10,543	10,543	10,543
R-squared	0.005	0.011	0.015			

^***^, ^**^, and ^*^ respectively indicate significance at the 1, 5, and 10% levels.

### Heterogeneity Test

In order to distinguish the impact of different types of samples on the regression results, this paper divides the samples according to the type of urban and rural areas, income status, area, whether they have completed compulsory education, and the age stage. Among them, urban and rural types and completion of compulsory education are both 0–1 dummy variables; urban and compulsory education are both assigned a value of 1; and rural and uncompleted compulsory education are both assigned a value of 0. Refer to existing literature practices (37), divide income into high-income and middle-income categories by 0.75 quintile, and assign west area = 1, central area = 2, and east area = 3 to the region. The age of residents is divided into youth (18–44 years old), middle aged (45–59 years old), and elderly (60 years old and above) according to the standards of the World Health Organization. The results are shown in Table 6.

**Table 6:** Heterogeneity test (1).

**Variable**	**Rural area**	**Urban area**	**Western area**	**Central area**	**Eastern area**
Exercise frequency	−0.019^***^	−0.020^***^	−0.026^***^	−0.007	−0.021^***^
	(−3.72)	(−4.18)	(−3.93)	(−0.98)	(−4.20)
Rural types	-	-	0.024	0.038	0.032
			(0.54)	(0.81)	(0.96)
Gender	−0.244^***^	−0.167^***^	−0.289^***^	−0.179^***^	−0.164^***^
	(−7.53)	(−5.93)	(−7.03)	(−4.00)	(−5.41)
Age	0.017^***^	0.015^***^	0.018^***^	0.014^***^	0.015^***^
	(12.06)	(12.43)	(10.55)	(7.20)	(11.22)
Marriage status	0.017	0.017	0.087	−0.118^*^	0.021
	(0.35)	(0.44)	(1.53)	(−1.67)	(0.49)
Party membership	−0.285	0.001	−0.134	0.314	−0.144
	(−1.22)	(0.01)	(−0.59)	(0.85)	(−0.80)
Education level	0.014	−0.009	0.001	−0.014	−0.000
	(0.83)	(−0.70)	(0.07)	(−0.64)	(−0.02)
Happiness level	−0.090^***^	−0.077^***^	−0.075^***^	−0.078^***^	−0.093^***^
	(−12.65)	(−11.08)	(−8.40)	(−7.16)	(−12.97)
Hospitalization experience	0.510^***^	0.455^***^	0.484^***^	0.518^***^	0.469^***^
	(11.04)	(10.26)	(8.59)	(7.83)	(9.69)
Chronic disease	0.727^***^	0.610^***^	0.704^***^	0.702^***^	0.635^***^
	(17.39)	(15.87)	(13.25)	(11.91)	(15.53)
Medical insurance	0.036	0.015	0.005	0.082	0.030
	(0.58)	(0.31)	(0.05)	(0.85)	(0.62)
Household income	−0.026^**^	−0.043^***^	−0.067^***^	−0.026	−0.025^**^
	(−1.96)	(−3.36)	(−3.71)	(−1.15)	(−2.01)
Household expenditure	0.007	0.008	0.027	0.025	−0.013
	(0.38)	(0.52)	(1.21)	(0.94)	(−0.87)
Family size	−0.013	−0.019^**^	0.001	−0.033^***^	−0.012
	(−1.42)	(−2.14)	(0.12)	(−2.58)	(−1.28)
Healthcare expenditure	0.054^***^	0.043^***^	0.045^***^	0.033^***^	0.054^***^
	(8.55)	(8.36)	(5.51)	(3.59)	(10.00)
Neighbor relations	−0.019^**^	−0.027^***^	−0.019^**^	−0.029^**^	−0.022^***^
	(−2.56)	(−3.72)	(−2.02)	(−2.56)	(−2.99)
Gift expenditure	0.096^*^	−0.028	0.160^**^	0.152^*^	−0.048
	(1.71)	(−0.61)	(2.03)	(1.83)	(−1.04)
_cons	2.758^***^	3.274^***^	2.793^***^	2.881^***^	3.267^***^
	(13.82)	(19.49)	(10.96)	(10.26)	(18.40)
N	5,243	5,300	2,929	2,491	5,123
R-squared	0.221	0.217	0.240	0.209	0.218

^***^, ^**^, and ^*^ respectively indicate significance at the 1, 5, and 10% levels.

Taking into account the basic national conditions of the dual structure of urban and rural areas in China, there are significant differences in lifestyles between rural residents and urban residents; the ways of participating in physical exercise are also different (13); and urban and rural income levels are different. Conversely, the allocation of medical resources is also different (8); the environmental indicators in rural areas and the green space that can be provided for physical exercise are also different from those in cities (21). So we will analyze the differences between urban and rural areas. From the perspective of urban–rural differences, exercise frequency is significant at the level of 1% for both urban and rural residents, but the absolute value of the regression coefficient of the urban sub-sample is slightly larger than that of rural residents. This shows that the increase in the degree of health of urban residents participate in exercise is more obvious. It may be due to the fact that rural residents often do physical work, and the effect of increasing participation in exercise to improve health is not obvious. Furthermore, standardized labor union organizations in cities can effectively guide urban residents to do physical exercise, but rural residents are still in a state where the pressure of survival is higher than the enjoyment (26).

Regional differences make differences in human factors such as economic development levels and health expenditures (23), and there are differences in sports venues and sports facilities. The scope of medical insurance in the west is gradually expanding, while the development level in the east is high, living conditions and quality are good, but the tight work life leads to lack of exercise (8). From the perspective of regional distribution, exercise frequency has a significant positive impact on the health of residents in the western and eastern regions at the level of 1% but has little impact on the health of residents in the central area. It is considered that residents in the economically developed eastern area have more ability to buy healthcare appliances to be used for physical exercise in more ways, while the economic conditions of the residents in the central area are slightly poorer, the conditions for physical exercise are insufficient, and there is a lack of exercise. So the degree of improvement in their health status is not large.

In addition, the level of income will also affect the level of health. The higher the level of income, the more likely it is to purchase health products, improve diet, and increase investment in fitness to improve physical health (8). From the perspective of income differences, the frequency of exercise significantly affects the health status of all income groups, the regression coefficient of a sample of high-income groups is relatively large in absolute value, and it has a greater impact on high-income groups. The results are shown in Table 7. It may be that middle-income residents are engaged in manual labor, so even if the frequency of participation in exercise by middle-income groups increases, the effect of improving health is not significant.

**Table 7:** Heterogeneity test (2).

**Variable**	**Middle income**	**High income**	**Compulsory education not completed**	**Complete compulsory education**
Exercise frequency	−0.015^***^	−0.032^***^	−0.022^***^	−0.016^***^
	(−3.67)	(−4.86)	(−3.98)	(−3.64)
Rural types	0.058^**^	−0.006	0.064^*^	0.043
	(2.19)	(−0.13)	(1.74)	(1.50)
Gender	−0.212^***^	−0.168^***^	−0.305^***^	−0.112^***^
	(−8.35)	(−4.39)	(−8.89)	(−4.22)
Age	0.015^***^	0.018^***^	0.014^***^	0.016^***^
	(14.07)	(10.94)	(9.36)	(14.17)
Marriage status	−0.002	0.060	−0.017	0.045
	(−0.06)	(1.01)	(−0.34)	(1.16)
Party membership	−0.113	−0.091	−0.487	−0.059
	(−0.64)	(−0.49)	(−1.02)	(−0.47)
Education level	−0.013	0.038^**^	-	-
	(−1.05)	(2.27)		
Happiness level	−0.086^***^	−0.078^***^	−0.079^***^	−0.090^***^
	(−15.27)	(−7.15)	(−10.76)	(−13.15)
Hospitalization experience	0.506^***^	0.391^***^	0.470^***^	0.489^***^
	(13.74)	(5.96)	(10.13)	(10.98)
Chronic disease	0.697^***^	0.561^***^	0.695^***^	0.630^***^
	(21.08)	(10.20)	(16.36)	(16.57)
Medical insurance	−0.001	0.064	0.011	0.038
	(−0.02)	(0.86)	(0.18)	(0.78)
Household income	-	-	−0.030^**^	−0.039^***^
			(−2.06)	(−3.23)
Household expenditure	0.001	0.022	0.002	0.008
	(0.07)	(1.01)	(0.13)	(0.57)
Family size	−0.010	−0.024^**^	−0.021^**^	−0.011
	(−1.33)	(−2.15)	(−2.20)	(−1.30)
Healthcare expenditure	0.048^***^	0.046^***^	0.067^***^	0.034^***^
	(10.16)	(6.10)	(9.89)	(6.88)
Neighbor relations	−0.025^***^	−0.015	−0.021^***^	−0.025^***^
	(−4.11)	(−1.45)	(−2.79)	(−3.56)
Gift expenditure	0.000	0.086	0.115^**^	−0.071
	(0.00)	(1.13)	(2.06)	(−1.54)
_cons	2.845^***^	2.040^***^	2.951^***^	3.188^***^
	(19.21)	(7.27)	(14.43)	(19.51)
N	7,901	2,642	4,755	5,788
R-squared	0.219	0.188	0.208	0.208

^***^, ^**^, and ^*^ respectively indicate significance at the 1, 5, and 10% levels.

Completion of compulsory education will also affect the participation rate of physical exercise to a certain extent. Studies have shown that educated people will do more exercises than those not educated in order to improve physical function and adjust mentality (9). In addition, compulsory education can improve people's years of education, thereby changing people's perception of physical exercise and health and prompting people to exercise (11). From the perspective of whether compulsory education has been completed, the compulsory education group sample's absolute value of the regression coefficient is relatively large, and it indicates that those who have completed compulsory education are less effective in improving their physical health through exercise than those who have not completed it. It may be that those with a higher education level have a more comprehensive understanding of health and will improve their health through other methods.

## Research Conclusions and Enlightenment

This paper uses the 2018 data of the CFPS, based on OLS and tobit models, to study the relationship between participation in physical exercise and the health of residents, and the following main conclusions are drawn: first, participation in physical exercise significantly promotes the health of residents. The higher the frequency of participation in exercise, the better the health status. Moreover, after a variety of robustness tests, the results are still consistent with the benchmark regression results. Second, the impact of participation in physical exercise on the health of residents has individual characteristics. Physical exercise has more obvious effects on youth and the elderly, those who have not completed compulsory education, and high-income groups. Third, there are regional differences in the impact of participating in physical exercise on the health of residents. The effect of physical exercise for urban residents in promoting health is better than that of rural residents, and the eastern and western areas are better than the central area.

The research in this paper not only confirms the rationality of enhancing physical fitness and improving mental health through physical exercise but also has reference significance and practical value for the formulation and implementation of policies. Regarding the improvement of residents' health, the state's requirements in the field of sports have ranged from nationwide fitness to sports power, demonstrating the powerful role of sports in promoting residents' health. Therefore, in order to promote all residents to improve their health through sports and to achieve the strategic goal of the construction of healthy China, this paper puts forward the following inspirations.

First, attach importance to residents' participation rate in sports and urge residents to incorporate sports into their lives. Promote a healthy lifestyle of physical exercise, enhance people's awareness of promoting health through exercise, combine sports health management with modern technology, promote sports into life, make people live in sports, and maintain a good state of participation in sports, thereby enhancing health level. Second, raise the income level of residents and pay attention to and guide young and old people to perform correct physical exercises. While consolidating the effectiveness of poverty alleviation, education should also be strengthened to cultivate talents to drive the development of industries in backward areas to increase residents' income. In addition, the knowledge of sports healthcare should be widely popularized to perform physical exercises correctly and effectively. Third, the sports resources are tilted toward the countryside, and the construction of sports facilities and venues in the central and western areas should be strengthened. Pay attention to the physical exercise of residents in backward areas, increase the construction of sports infrastructure in rural areas and the central and western areas, send sports talents to the countryside, and correctly guide rural residents to exercise.

## Data Availability Statement

The original contributions presented in the study are included in the article/supplementary material, further inquiries can be directed to the corresponding author/s.

## Author Contributions

CL: conceptualization, methodology, and software. J-HC: data curation, writing-original draft preparation. X-HL: visualization and investigation. S-QR: writing-reviewing and editing. All authors contributed to the article and approved the submitted version.

## Conflict of Interest

The authors declare that the research was conducted in the absence of any commercial or financial relationships that could be construed as a potential conflict of interest.
